# The Saga of Endocrine FGFs

**DOI:** 10.3390/cells10092418

**Published:** 2021-09-14

**Authors:** Phuc Phan, Bibhuti Ballav Saikia, Shivakumar Sonnaila, Shilpi Agrawal, Zeina Alraawi, Thallapuranam Krishnaswamy Suresh Kumar, Shilpa Iyer

**Affiliations:** 1 Department of Chemistry and Biochemistry, Fulbright College of Art and Sciences, University of Arkansas, Fayetteville, AR 72701, USA; phucphan@uark.edu (P.P.); sksonnai@uark.edu (S.S.); sagrawal@uark.edu (S.A.); ziibrahe@uark.edu (Z.A.); 2 Department of Biological Sciences, Fulbright College of Art and Sciences, University of Arkansas, Fayetteville, AR 72701, USA; saikia@uark.edu

**Keywords:** fibroblast growth factors, FGF19, FGF21, FGF23, endocrine FGFs, klotho, FGFR, cell signaling, biomedical applications, metabolic disease

## Abstract

Fibroblast growth factors (FGFs) are cell-signaling proteins with diverse functions in cell development, repair, and metabolism. The human FGF family consists of 22 structurally related members, which can be classified into three separate groups based on their action of mechanisms, namely: intracrine, paracrine/autocrine, and endocrine FGF subfamilies. FGF19, FGF21, and FGF23 belong to the hormone-like/endocrine FGF subfamily. These endocrine FGFs are mainly associated with the regulation of cell metabolic activities such as homeostasis of lipids, glucose, energy, bile acids, and minerals (phosphate/active vitamin D). Endocrine FGFs function through a unique protein family called klotho. Two members of this family, α-klotho, or β-klotho, act as main cofactors which can scaffold to tether FGF19/21/23 to their receptor(s) (FGFRs) to form an active complex. There are ongoing studies pertaining to the structure and mechanism of these individual ternary complexes. These studies aim to provide potential insights into the physiological and pathophysiological roles and therapeutic strategies for metabolic diseases. Herein, we provide a comprehensive review of the history, structure–function relationship(s), downstream signaling, physiological roles, and future perspectives on endocrine FGFs.

## 1. Introduction

The fibroblast growth factor (FGF) family comprises twenty-two members that share a similar core protein sequence and structure, but each subfamily exhibits a wide range of biological functions [1]. Their molecular masses range from 17 kDa to 34 kDa [2]. The FGF family is further classified based on their sequence homology and phylogeny into seven subfamilies: FGF1, FGF4, FGF7, FGF8, FGF9, FGF11, and FGF19 subfamilies [3]. The seven subfamilies possess different modes of action (autocrine/paracrine, intracrine, and endocrine) [2,4]. Paracrine/autocrine FGFs (FGF1, FGF4, FGF7, FGF8, and FGF9 subfamilies) assert their functions locally through interaction and activation of cell surface tyrosine kinase FGF receptors (FGFRs) through a high-affinity interaction with heparin or heparan sulfate [5,6,7,8]. The human *FGFR* family comprises four members: FGFR1–FGFR4. Although all the FGFRs are encoded by distinct genes, the sequence of the four members bears high similarity (56% to 71%) [9]. Structurally, the FGFRs resemble receptor tyrosine kinases (RTKs) with an extracellular ligand-binding domain at the N-terminus followed by a single-pass transmembrane domain, and a split cytoplasmic tyrosine kinase domain at the C-terminus [2]. The extracellular ligand-binding domain consists of three immunoglobulin domains (D1-D3). D1 and D2 domains are responsible for the receptor autoinhibition and the ligand-receptor binding, respectively. Both the D1 and D2 domains are connected through a sequence rich in aspartic acids, termed as acid box. D2-D3 domains are responsible for ligand-binding and specificity [3]. *FGFR1-3* genes undergo an alternative splicing event in the D3 domain to yield two alternative splicing variants (b and c). These splice variants have distinct ligand-binding specificities. Thus, there are seven FGFR variants: FGFR1b, FGFR1c, FGFR2b, FGFR2c, FGFR3b, FGFR3c, and FGFR4 [9]. The paracrine subfamilies regulate a variety of cellular and developmental processes such as: brain patterning, branching morphogenesis, limb development, cellular proliferation, survival, and migration [7,10,11]. On the other hand, members of the endocrine FGF subfamily (FGF15/19, 21, and 23; FGF15 is a mouse ortholog of FGF19) act as endocrine-like secretions [9,12]. They mediate physiological processes through FGFR interactions, but over longer range such as hormones, do not solely require heparin or heparan sulfate [4,9]. However, the endocrine FGFs require other co-receptors, called klotho, for binding and activation of FGFRs [4,9]. Therefore, these FGFs bind to the FGFR–Klotho complex. The C-terminus of FGF19, 21, and 23 contains the binding site for the FGFR–Klotho complex. β-Klotho (KLB) augments the FGF19 signaling by binding to FGFR4, and FGF21 to FGFR1c, whereas FGF23 activates the signaling complex composed of FGFR1c and α-Klotho (KLA) [9]. They have evolved to regulate metabolisms of bile acids, phosphates, carbohydrates, and lipids in addition to their canonical FGF functions [3,5]. Lastly, the FGF11 subfamily consists of FGF11–14 and functions only in the intracrine mode of action [7]. The intracrine FGFs, also called iFGF, are intracellularly expressed, and function through FGFR-independent pathways [4]. Members of the FGF11 subfamily are usually involved in the regulation of voltage-gated sodium channels [4]. Mutations in FGFRs in humans are involved in various pathologies ranging from different forms of cancers and skeletal disorders [10].

In contrast to paracrine FGFs, members of the endocrine FGFs subfamily have unusually poor affinities for their cognate FGF receptors and require KLA and KLB co-receptor proteins [7,8]. Out of the three members (FGF19, FGF21, and FGF23), FGF 19 shows a higher degree of heparin-dependent downstream signaling [13]. Endocrine FGFs are critical for maintaining homeostasis of the whole organism due to their various roles in survival, development, metabolism, and its adaptations, as well as modulation of vitamin D and phosphate levels [6]. Given the plethora of functions, the endocrine FGFs subfamily has been a desired target for therapeutic development [6,10,14,15,16,17,18,19,20]. In this review, we attempt to provide a comprehensive account of the progress in the research on the structure, signaling, physiological roles, and therapeutic applications of endocrine FGFs.

### 1.1. Endocrine FGFs—A Historical Perspective

#### 1.1.1. Discovery of FGF19

**Figure 1:**
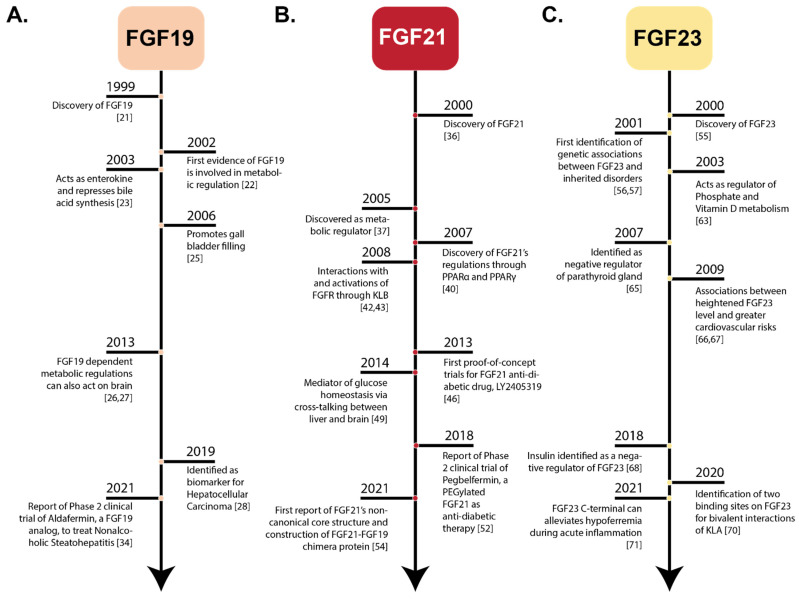
Historical Timelines of Endocrine FGFs. Historical perspective of notable events in the discovery and development of endocrine FGFs. (Panel-**A**): FGF19 timeline from discovery to the most recent clinical trial for FGF19-based biomedical application, aldafermin. (Panel-**B**): FGF21 timeline from discovery to the most recent findings pertaining to the structure of FGF21. (Panel-**C**): FGF23 timeline from discovery to the most recent findings regarding its therapeutic role in treating acute inflammation.

Since the first identification of the acidic FGF (FGF1) in 1976, the FGF family has grown in importance into a 22-member protein family with a myriad of biological functions [1]. Likewise, information and studies regarding members of the endocrine subfamily have also been growing steadily, ever since the discovery of FGF19 in 1999 and later discoveries of FGF21 and FGF23 in the year 2000 (Figure 1). FGF19 was first identified in the fetal brain and was thought to play a key role in early neuronal development [21]. In 2002, the first involvement of FGF19 in metabolic regulation was revealed when transgenic mice expressing FGF19 showed increased metabolic rate and decreased adiposity [22]. In 2003, FGF19 was found to be involved in the feedback repression of bile acid biosynthesis pathways [23]. In 2006, FGF19 was shown to inhibit the action of Cholecystokinin or CCK, an intestinal postprandial hormone known to empty the gallbladder, directly through FGFRs [24]. These data suggest that FGF19 plays an important factor in regulating the cycle of bile acid; from biosynthesis to filling and emptying; thus, delineating the hallmark of its physiological function (Figure 1A). FGF19 was also found to regulate systemic metabolism by acting on the brain [25,26]. Administration of FGF19 directly into the brain of obese *ob/ob* mice improved glucose tolerance within two hours through its action in the brain [26]. Interest in studying FGF19 increased due to its ability to act as a potential biomarker with high sensitivity for hepatocellular carcinoma (HCC) [27,28]. Emerging data in HCC studies implicate that aberrant expression of FGF19 and FGFR4 lead to tumor proliferation, thereby causing HCC [29,30,31,32]. Indeed, several studies show that the increased serum levels of FGF19 have high sensitivity and selectivity for HCC [27,28]. Continued research on FGF19 as therapeutic agents resulted in the design of NGM282, which is also called Aldafermin, an engineered FGF19 analog that was used in phase 2 human safety and efficacy trials to treat patients diagnosed with non-alcoholic steatohepatitis (NASH) [33] (Table 1). After 24 weeks, NASH patients treated with Aldafermin showed a significant reduction in liver fat content with a trend towards fibrosis improvement [33] (Table 1). Administration of Aldafermin to patients with metabolic/cholestatic liver diseases showed that the drug suppressed the glycine-conjugated hydrophobic bile acids, which have high cytotoxicity and detergent activity, and allowed the utilization of Aldafermin in treating gastrointestinal and liver conditions [34].

#### 1.1.2. Identification of FGF21

FGF21 was discovered in humans and mice via a polymerase chain reaction approach using FGF-derived degenerative primers and the highest expression of FGF21 transcript was found in mouse liver and thymus [1,35]. FGF21 was demonstrated to share 75% amino acid identity between mice and humans [35]. Studies showed that secretion of FGF21 was controlled by metabolic signaling pathways such as peroxisome proliferator-activated receptor γ (PPARγ) [36] and peroxisome proliferator-activated receptor α (PPARα) [37,38], two key nuclear transcription regulators that affect target genes involved in glucose and lipid metabolism, cell proliferation, differentiation, and immune responses [36,37,38,39]. While it was proposed that FGF21 may require a specific co-factor to activate FGFR proteins, its specific interaction with KLB was demonstrated much later to enable FGF21-mediated activation of FGFRs in vitro and in vivo [1,36,37,38,39,40,41,42]. The biological activity of FGF21 was documented wherein recombinant FGF21 stimulated glucose incorporation in human and mouse adipocytes [40]. Furthermore, when FGF21 was overexpressed in obese and diabetic models of transgenic mice, it led to decrease of glucose levels in an insulin-independent manner, thus marking the protein’s therapeutic potential [40]. Detailed interaction mechanism between the C-terminus of FGF21 and its receptors and co-receptors was discovered in late 2008, further aiding the structure-based optimization of FGF21 [43,44] (Figure 1B). The first major optimization for FGF21 came from Lilly Laboratory in 2013, called LY2405319, a variant with comparable bioactivity to native FGF21, and improved biophysical characteristics [45]. The first proof-of-concept trials for LY2405319 were reported in both diabetic monkeys and human subjects [46,47]. The results of the trial for LY2405319 and its glucose uptake effects, however, were less robust in human subjects than rodents and monkeys, despite other positive outcomes such as lipid, insulin, and body weight measures [1]. Specifically, administration of LY2405319 reduced body weight, insulin level, and triglycerides level in humans, mice, and monkeys [1,46,47]. LY2405319 also decreased the levels of low-density lipoprotein, cholesterol, and increased the high-density lipoprotein cholesterol in monkeys and humans [1,46,47]. FGF21 has also been reported to play a crucial role in maintaining glucose homeostasis by facilitating inter-organ crosstalk between the liver and brain [48]. In this study, fasting FGF21 knock-out mice displayed severe hypoglycemia and impaired hepatic gluconeogenesis [48]. These problems were reversed when the FGF21 knock-out mice were provided intracerebroventricular injections of exogenous recombinant FGF21 (rmFGF21) [48]. Furthermore, reversal effects of FGF21 in FGF21 knock-out mice were abrogated by blocking the hypothalamic FGFR1 [48]. During fasting, the transcription factor, PPARα, is activated in the liver [49]. Like FGF21 knockout mice, PPARα knockout mice exhibited similar phenotypes such as hypoglycemia and impaired hepatic gluconeogenesis [36,37,38,39,48]. Intracerebroventricular administration of rmFGF21 into PPARα knock-out mice significantly reversed the hypoglycemic phenotypes [48]. The results indicate that FGF21 mediates glucose homeostasis through PPARα which is a master regulator coordinating metabolic adaptions to fasting and starvation [48]. Recently, phase-2 clinical trials for Pegbelfermin, a PEGylated FGF21, reported this agent to be an effective treatment for NASH patients [50,51]. On the other hand, Pegbelfermin as an agent for type-2 diabetes mellitus (T2DM) showed no change in body weight or glycated hemoglobin, but improved insulin sensitivity, triglycerides, and high-density lipoprotein cholesterol levels [52]. In 2021, a study reported the core structure of FGF21 for the first time [53]. Using the newly discovered core structure of FGF21, Zhu et al. (2021) designed a structure-based FGF21 therapeutic drug that can potentially decrease blood glucose levels in diabetic and obese mice [53]. Thus, this discovery opens new opportunities for structurally-based therapeutic agents of FGF21 for selective metabolic disorders such as T2DM and obesity [1]. 

#### 1.1.3. History of FGF23

The third member of the endocrine subfamily, FGF23, was first identified from mouse embryos by homology-based polymerase chain reaction [54]. By the homology search approach, the human *FGF23* gene was found to be highly similar (72% amino acid identity) to mouse *FGF23* [54]. In 2001, for the first time, genetic associations between FGF23 and inherited disorders were reported [55,56] (Figure 1C). For example, the *FGF23* gene with a gain of function mutations may cause autosomal dominant hypophosphatemic rickets (ADHR) [55,56]. This mutation resulted in the secretion of an FGF23 variant, which is less sensitive to protease cleavage than the wild-type FGF23. Therefore, due to the resistance against proteolysis, an elevated level of circulating FGF23 leads to phosphate wasting in ADHR patients [55,56]. Phosphate is an essential nutrient in all processes of energy metabolism and skeletal mineralization [57]. It is an important source of the organic constituent of DNA, RNA, and phospholipids in the cell membrane and is essential for ATP formation mainly through energy metabolism or cellular signaling modulators [58]. Given its importance in living organisms, FGF23 was found to be a phosphaturic protein that is primarily secreted by bone to maintain phosphate levels in response to Vitamin D [59,60]. FGF23 derived from bone acts on kidneys to regulate phosphate reabsorption and vitamin D production, representing an important endocrine loop in the control of mineral homeostasis [61]. In vivo studies revealed FGF23 to be a unique regulator of both phosphate and vitamin D metabolism via less understood mechanisms [62,63]. Injection of recombinant FGF23 led to reduced serum phosphate and 1,25(OH)2D (dihydroxy vitamin D) levels in mice [62]. Additional changes in mRNA levels of the key enzymes of the vitamin D metabolism were also observed after the injection of FGF23; leading to an increase in 25-hydroxyvitamin D-24-hydroxylase mRNA levels and a decrease in 25-hydroxyvitamin D-1α-hydroxylase mRNA levels [62]. FGF23 was subsequently identified as a negative regulator of parathyroid hormone (PTH) expression, which is a novel function of FGF23 along with other well-established functions in controlling vitamin D and phosphate metabolism [64]. Several reports show that elevated levels of FGF23 are associated with increased risk of left ventricular hypertrophy and increased left-ventricular mass index in elderly subjects, both with chronic kidney disease (CKD) patients and healthy subjects [65]. Both measures contributed to a high risk for increasing cardiovascular mortality in CKD [65]. Additionally, higher FGF23 serum levels were also associated with higher total body atherosclerosis [66]. Together, the data suggested a deeper relationship between FGF23 and cardiovascular risks that warrant further interest. Insulin has been a direct negative regulator of FGF23 [67]. The mechanism involved in the inhibition of FGF23 production by insulin and insulin-like growth factor 1 (IGF1) was mediated through phosphoinositide 3-kinase (PI3K)/protein kinase B (PKB/Akt)/ forkhead box protein O1 (FOXO1) signaling [67]. Interaction between FGF23 and KLA suggested that the C-terminus of FGF23 has two binding sites for KLA enabling a bivalent interaction [68,69]. Each of these binding sites was shown to bind to KLA on its own with a similar binding affinity [68,69]. Another recent study has demonstrated that inhibition of FGF23 signaling alleviates hypoferremia during acute inflammation [70]. 

Current studies on the endocrine FGF family have demonstrated the importance of FGF19, FGF21, and FGF23 in human genetic and metabolic disorders, suggesting their therapeutic role in metabolic diseases (Figure 1).

## 2. Physiology and Clinical Significance of Endocrine FGFs

The history of endocrine FGFs provides a strong indication of the interest of researchers toward investigating the structure-function relationship of these important classes of proteins and allows ongoing research in the field to expand rapidly. The progress of research on the physiological roles of endocrine FGFs is highlighted here, with a focus on their clinical significance.

### 2.1. Physiology of FGF19 

**Figure 2:**
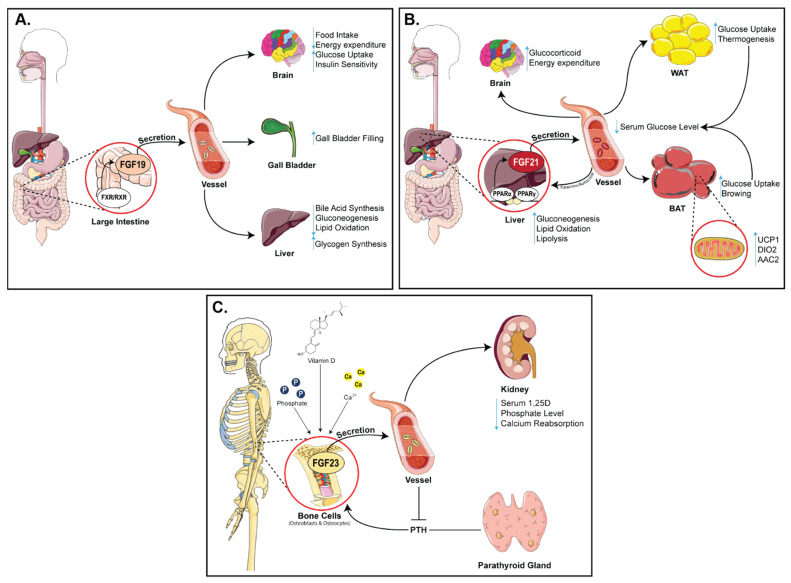
Physiological Activities of Endocrine FGFs. (Panel-**A**): FGF19 is excreted from the large intestines and acts upon different organs such as the brain, gall bladder, and liver to perform important functions such as regulation of bile acid biosynthesis and gall bladder filling. In the liver, FGF19 suppresses the synthesis of bile acid and decreases gluconeogenesis and lipid oxidation, whereas it increases glycogen synthesis. In the brain, FGF19 inhibits food intake, decreases energy expenditure, and increases glucose tolerance. (Panel-**B**): FGF21 is expressed in the liver and acts upon the brain, white adipose tissues, and brown adipose tissues to regulate glucose uptake. FGF21 also acts in a paracrine/autocrine manner to regulate gluconeogenesis and lipid oxidation. It stimulates the expression of mitochondrial UCP1 in white adipose tissue (WAT) and increases glucose uptake in brown adipose tissue (BAT). (Panel-**C**): FGF23 is expressed in the bone marrow and mainly acts upon the kidney to regulate phosphate and calcium levels. FGf23 is also involved in regulation of PTH levels, lowering the levels of serum phosphate and 1,25 dihydroxyvitamin D.

FGF19 is involved in the organogenesis of energy-intensive organs such as the ear, eye, heart, and brain [71,72]. FGF19 is also expressed in human embryonic stem cells and the expression levels correlate with the pluripotent state [73]. In contrast, during adulthood, FGF19 would disappear globally from the central nervous system and is predominantly expressed in the ileum, and acts in the human liver through its receptor FGFR4 and co-receptor KLB [73]. The binding of FGF19 to FGFR4 leads to phosphorylation of extracellular signal-regulated protein kinase 1 (ERK1), extracellular signal-regulated protein kinase 2 (ERK2), and Stat-3, and this affects the proteins involved in the regulation of gluconeogenesis, lipogenesis, bile acid biosynthesis, and cell proliferation [74,75,76] (Figure 2A). Bile acids are important for intestinal lipid absorption and their synthesis represents a major pathway for cholesterol catabolism [77]. In physiological states, circulating levels of FGF19 represses bile acids synthesis, which are signaling molecules that activate specific bile salt receptors (FXR) during enterohepatic circulation from the ileum to the liver [78]. FXR is a bile acid nuclear receptor encoded by Nuclear Receptor Subfamily 1 Group H Member 4 (*NR1H4)* gene [11,23]. Specifically, bile acid activates FXR and subsequently upregulates the synthesis of FGF19 [79]. In turn, FGF19 creates a negative feedback loop and negatively regulates the bile acid biosynthesis by suppressing the expression of Cholesterol 7a-hydroxylase (CYP7A1), a rate-limiting enzyme in the production of bile acid [80]. A study showed that in FXR-deficient mice, FGF19 cannot be expressed, resulting in an elevated expression of CYP7A1. This indicates that the bile acid synthesis is abolished [81]. However, recent studies suggest that there may be an FGF19-independent suppression of bile acid synthesis through the activation of hepatic FXR [82] creating new opportunities for further investigation on the role(s) of FGF19 in bile acid regulation. FGF19 is also expressed in human epithelial cells of the gallbladder. Bile acid is stored in the gallbladder until it is needed by the body [83]. FGF19 is expressed due to the bile acid-mediated induction of FXR. The expression of FGF19 subsequently induces gallbladder relaxation and filling [80]. Additionally, another postprandial hormone involved in the gallbladder emptying process, CCK, is also affected by FGF19 [84]. Specifically, CCK aids in the emptying process of the gallbladder, which stores and concentrates bile acid during fasting, and releases bile acid during digestion [83,84]. FGF19 administration suppresses the action of CCK directly which leads to the induction of gallbladder filling in CCK-treated mice [24,80].

Studies in mice with whole-body FGFR4 deficiency have impaired glucose tolerance and reduced insulin sensitivity [85]. Treatment with FGF19 regained the normal glucose tolerance. These findings provided a piece of evidence for a new physiological role of FGF19 in glucose homeostasis [5,80]. FGF19 contributes to the regulation of hepatic glucose homeostasis through its effect on gluconeogenesis in an insulin-independent manner [14]. More specifically, FGF19 represses the expression of a gluconeogenic gene through cAMP-response element-binding/protein-peroxisome proliferator-activated receptor-γ co-activator 1α (CREB/PGC1α) pathway that is not dependent upon insulin signaling [14]. This allows FGF19 to activate ERK 1 and 2 signaling in adipocytes and increase glucose uptake [81,86]. Furthermore, FGF19 can promote glycogen synthesis to further regulate glucose homeostasis [80]. Glycogen synthesis is negatively regulated by glycogen synthase kinase 3α (GSK3) α and β, i.e., these enzymes phosphorylate and inactivate glycogen synthase to suppress glycogen synthesis [80]. However, FGF19 induces the inactivation of GSK3α and GSK3β thereby, increasing glycogen synthesis [80]. With evidence that FGF19 is a key regulator of metabolism, further interests in the field of FGF19 reported a new function for the protein in skeletal muscle. Previous works showed that mTOR and its downstream target, ribosomal protein S6 kinase (S6K), are the key regulators of muscle cell growth [87,88]. Furthermore, it has been reported that mice treated with recombinant FGF19 consecutively for 7 days had a higher skeletal muscle mass and strength than the control mice [89]. Additionally, treatment with recombinant FGF19 increased the myotube area in human muscle cells during differentiation from myoblast to myotube, as well as in fully differentiated myotube [89]. Recently, Guo et al. (2021) demonstrated the role of FGF19 in regulating skeletal muscle of mice by enlarging muscle fiber size as well as detailed a mechanism for such function [90]. Specifically, FGF19 increases the muscle fiber size by stimulating the phosphorylation of the ERK 1 and 2 and S6K1 pathways [90]. Furthermore, they also showed that FGF19 was able to protect the skeletal muscle against obesity-induced muscle atrophy through the AMP-activated protein kinase (AMPK)-Sirtuin-1 (SIRT1)-PGC1α pathway, which may be a potential target for obesity treatment [90]. 

In addition, recent findings indicate that FGF19 might have an interesting relationship with mitochondrial function(s) through the mediation of the palmitate oxidation process [91]. The excess levels of palmitate had been shown to inhibit glucose uptake, increase insulin resistance, and upregulate β-oxidation in mitochondria leading to mitochondrial overload accompanied by increased levels of reactive oxygen species (ROS) production [92,93]. FGF19 was recently found to promote the expression of mitochondrial biogenesis and antioxidant response regulators (PGC1α, TFAM, HO-1, etc.) in skeletal muscle [91]. The regulatory effects of FGF19 are postulated to involve the AMP-activated protein kinase (AMPK)/PGC1α pathway to help amend the palmitate-induced mitochondrial dysfunction in skeletal muscle [91]. Similarly, a recent study reported that treatment of FGF19 prevented mitochondrial overload and apoptosis while maintaining normal insulin signaling in mouse myoblasts (C2C12) cells with palmitate-induced dysfunction [93]. Consequently, the effects of palmitate led to activation of the mitochondria-mediated apoptosis process, inhibition of insulin signaling pathway, and myotube atrophy [92,93]. However, administration of FGF19 facilitated inhibition of the β-oxidation of palmitate, consequently alleviating mitochondrial overload and prevention of mitochondria-mediated apoptosis [93]. 

Another new area of research for FGF19 is its ability to induce protein synthesis by increasing the phosphorylation of eukaryotic initiation factor 4B (eIF4B) and eukaryotic initiation factor 4E (eIF4E). Both these initiation factors are part of the eIF4F complex that induces the binding of mRNA to the ribosome [80]. Furthermore, FGF19 increases the phosphorylation of the ribosomal protein subunit 6 (rpS6) and enhances protein synthesis [7]. These new areas give a better grasp of the diverse role of FGF19 in the system and may provide clues for future therapeutical developments. 

### 2.2. Clinical Significance of FGF19 

FGF19 is a metabolic regulator and is associated with many diseases. Studies have demonstrated that an elevated level of serum FGF19 is observed in CKD patients and is associated with lipid and carbohydrate metabolism [94]. Moreover, the study also indicated that hemodialysis and kidney transplantation result in the reduced levels of FGF19 [94]. However, the causative factor for the increase in serum FGF19 concentration in CKD patients is still unknown. Additionally, FGF19 is believed to play a role in alcoholic hepatitis. In a recent study, it was shown that the serum levels of FGF19 were significantly increased, and the hepatic expressions of the *FGF19* gene were also induced in alcoholic hepatitis patients [95]. FGF19 is also associated with type 2 diabetes mellitus (T2DM). T2DM patients often suffer from atherosclerosis, thus, understanding the underlying mechanisms involved in metabolism is crucial in understanding atherosclerosis, which may ultimately improve the development of T2DM [96,97]. Subclinical atherosclerosis is an early indicator of the onset of atherosclerosis, which could be examined by increased intima-media thickness (IMT) of arteries [98]. A recent study found that serum levels of FGF19 were positively correlated with carotid IMT in men that showed that the level of FGF19 could be a predictor of subclinical atherosclerosis in men with [97]. Additionally, reports showed that the administration of NGM282, an analog of FGF19, has proven to be a protective factor for atherosclerosis [99]. Thus, FGF19-based therapeutic agents have the potential to be further developed to improve T2DM prognosis. Diabetic cardiomyopathy is another major cause of mortality in diabetic patients in which high cardiac oxygen consumption and mitochondrial dysfunction often result in ventricular dysfunction and cardiomyocyte death [100,101]. Recent works reported that FGF19 enhanced mitochondrial function through an increase in the expression of mitochondrial biogenesis regulators, i.e., PGC1α, via the AMPK/ NF-E2-related factor 2 (Nrf2)/HO-1 pathway, thus, relieving the oxidative stress on the heart and improved diabetic cardiomyopathy [91]. Despite the positive initial findings, the roles of FGF19 in regulating mitochondrial biogenesis and antioxidant responses are still under-studied and worthy of more investigation.

Interestingly, mitochondrial dysfunction has been shown to arise in many cases of non-alcoholic fatty liver diseases (NAFLD) and promotes liver damage, induces inflammation and fibrosis, and produces a hostile microenvironment that promotes anti-apoptotic programs and mutations of hepatocytes, leading to NAFLD-driven malignant hepatocytes and HCC [102]. With FGF19 having a new and detailed relationship with mitochondria and its functions as previously discussed, one can assume that FGF19 may also be intricately linked to cancers [27,91]. Indeed, as mentioned previously in the historical perspective of FGF19, the protein was found to be heavily involved with HCC and shown to be useful for the diagnosis and prognosis of the condition [27]. One study also proposed FGF19 as a potent marker and potential driver of lung squamous cell carcinoma (LSQ) [103]. Later, studies by the same group demonstrated for the first time that overexpression of FGF19 enhanced tumor cell proliferation and metastasis in an in vivo model of LSQ [104]. The results also showed that inhibiting the mechanistic target of rapamycin (mTOR) in the in vivo LSQ models overexpressing FGF19, was an effective way to inhibit the tumor. Thus, growing evidence indicates that the FGF19-FGFR4 pathway plays a vital role in the initiation and progression of distinct types of cancers [27,105,106]. However, the exact mechanism by which FGF19 influences the development of cancer remains unknown and requires additional studies. Such studies will likely trigger new interests in targeting FGF19-FGFR4 pathways with new or existing drugs and pave the way for new treatment options in various cancers. 

### 2.3. Physiology of FGF21

Experimental evidence suggests that FGF21 is a potent metabolic regulator of glucose uptake through stimulation of glucose transporter (GLUT) 1-mediated glucose uptake in 3T3L1 adipocytes [40] (Figure 2B). In addition to the role of FGF21 in the liver, this protein also acts in an endocrine manner in adipose tissues to stimulate lipolysis through PGC1α, the major regulator of mitochondrial functions and biogenesis [11,49]. Adipose tissue is classified into two parts: white adipose tissue (WAT), which has a role in energy and lipids storage, and brown adipose tissue (BAT), which functions in generating heat during the adaptive response to cold exposure [107]. In WAT, FGF21 is a potent inducer of uncoupling protein 1 (UCP1) which is mediated through PGC1α activity, and finally triggers thermogenic genes such as *type II iodothyronine deiodinase (DIO2)*, *PGC1α*, and *PPARα* [108,109]. Cold exposure enhances circulating FGF21 levels in humans, which corresponds to increased energy expenditure and lipolysis [110]. Mitochondrial dysfunction has been implicated in several neurodegenerative disorders like Parkinson’s disease, and Alzheimer’s disease [111]. Since PGC1α has been suggested as a therapeutic target to increase mitochondrial efficacy, studies demonstrate that FGF21 can enhance mitochondrial function by increasing mitochondrial antioxidants in human dopaminergic neurons, via NAD(+)- dependent deacetylase Sirtuin-1 (SIRT1) [112]. The proposed mechanism for such enhancement is through an increase in the phosphorylation of AMPK+, which increases cellular NAD+ levels leading to activation of SIRT1 and deacetylation of PGC1α, which in turn, increases oxygen consumption and citrate synthase activity in adipocytes [113].

FGF21 is an important regulator of macronutrient intake. It has been observed that fructose or alcohol consumption robustly increases plasma FGF21 [114,115]. Similarly, a high protein restriction or high carbohydrate diet increases FGF21 levels [116]. In addition, one study indicated that FGF21 is a liver-induced endocrine FGF that enters the circulation to act on the brain to suppress simple sugar intake and sweet-taste preference without altering the protein intake [117]. However, the mechanism underlying these effects has not been reported clearly. FGF21 signaling to glutamatergic neurons in the ventromedial hypothalamus (VMH) is required to regulate sugar intake [86]. Hill et al. (2017) recently demonstrated that FGF21 is essential for coordinating the metabolic response to protein restriction in lean mice [118]. Later, the same group reported that FGF21 signaling in the brain is only required to drive the metabolic response to protein restriction [119]. 

The previous reports indicated that FGFs are the active factors promoting hair follicle growth and development [120]. However, the relationship between FGF21 expression and hair follicle growth and development has not been reported well. A recent study demonstrated that FGF21 affects hair follicle growth and development which may be associated with PI3k/Akt and mitogen-activated protein kinase (MAPK)/ERK signaling pathways [121]. 

### 2.4. Clinical Significance of FGF21

It has been observed that the level of FGF21 fluctuates with different metabolic disorders. Indeed, elevated levels of FGF21 have been observed in patients with obesity and T2DM relative to lean subjects [122]. Additionally, Zhen et al. (2016) reported enzymes that facilitate enzymatic cleavage on FGF21 [123]. The first enzyme is dipeptidyl peptidase IV, which cleaves FGF21 at the N-terminus after the proline-2 and -proline-4 [123]. The second is fibroblast activation protein (FAP), which catalyzes the cleavage of FGF21 at the C-terminus end after proline 171, rendering FGF21 inactive [123]. Thus, inhibition of FAP is thought to be a potential approach to increase endogenous FGF21 activity for the treatment of obesity and T2DM [123]. Likewise, it has been well studied that the level of FGF21 is high in NAFLD patients [124]. Additionally, a recent study demonstrated that an FGF21-to-adiponectin ratio (FAR) is elevated in NAFLD children [125]. Therefore, FGF21 and adiponectin ratio could be a potentially useful biomarker of NAFLD in children [125]. 

FGF21 is a serum biomarker for several primary mitochondrial disorders (PMD) with high sensitivity and accuracy [126,127,128,129]. A recent meta-analysis of the literature which included five randomized controlled trials encompassing 718 FGF21 assessments demonstrated the utility of FGF21 as a promising biomarker for mitochondrial disease diagnosis [130]. The proposed mechanism of action regulating levels of FGF21 was by activating the mTOR-PGC1α pathway in PMD patients [131]. Another recent PMD study demonstrated that the mitochondrial integrated stress response is regulated by the autocrine and endocrine effects of FGF21 to induce step-wise changes in glucose and lipid metabolism, weight loss, and brain defects, emphasizing the importance of FGF21 as a key mediator of metabolic remodeling and progression of mitochondrial integrated stress response in PMDs [132].

The role of FGF21 in carcinogenesis is not well understood. Previous works reported an elevated serum level of FGF21 in aggressive thyroid cancer yet cannot precisely determine the origin of this upregulation of FGF21 [133]. Similarly, increased serum levels of FGF21 are also observed in breast cancers [134], renal cancer [135], and endometrial cancer [136]. These reports all indicate that FGF21 may be a suitable biomarker for diagnosis and prognosis for several types of cancers [133,134,135,136]. On the other hand, other studies have indicated that administration of recombinant FGF21 does not stimulate tumor growth in obese and diabetic patients [46,133,137], providing inconsistencies with other findings [136]. Such limited understanding of the role of FGF21 and detailed mechanism in cancer development may provide new avenues for future research.

### 2.5. Physiology of FGF23

FGF23 is a regulator of phosphate homeostasis and is synthesized by osteoblast, osteocyte, and bone marrow [138,139] (Figure 2C). It functions as a regulator of PTH metabolism through MAPK (ERK 1 and 2 and Akt) pathway [140]. The other key role of FGF23 is to suppresses the synthesis of 1α, 25-dihydroxy vitamin D3 (1,25(OH)2D3) in the kidney [62], whereas PTH and 1,25(OH)2D3 regulate the production of FGF23 [141]. A recent study reported insulin and IGF1 as negative regulators of FGF23 production. The suppression of FGF23 production by insulin and IGF1 was mediated through phosphoinositide 3-kinase (PI3K)/protein kinase B (PKB)/Akt/ forkhead box protein O1 (FOXO1) signaling [67]. FGF23 also has physiologically relevant functions in bone mineralization through FGFR3, in a klotho-independent pathway [142]. Tissue non-specific alkaline phosphatase (TNAP) plays a key role in the regulation of bone mineralization by cleaving the mineralization inhibitor, pyrophosphate, which is secreted by osteoblasts [143]. However, FGF23 is reported to be a powerful suppressor of transcription of TNAP mRNA in bone cells, which decreases the degradation of pyrophosphate and lowers the production of inorganic phosphate, leading to impaired bone mineralization [142]. 

A recent study identified that erythropoietin (EPO) could be a potential link between anemia, iron status, and FGF23 production [144]. An association between EPO and FGF23 was examined in healthy humans. It was found that EPO induced an increase in the levels of the C-terminus fragment of FGF23 (cFGF3) but not intact FGF23 (iFGF23) [144]. However, in mice, it has been observed that EPO first elevates C-term FGF23 and then iFGF23 [145]. It has been demonstrated that FGF23 controls the production of Fetuin A (ASGH), a serum protein with anti-calcification activity in osteocytes [146]. In addition, another study also identified the involvement of FGF23 in the regulation of Fetuin A in the liver with a biphasic effect [147].

Although the expression of FGF23 was identified in skeletal muscle tissue, its relationship with the skeletal muscle and mitochondrial functions merits further investigation [148,149]. However, a recent study demonstrated that physical exercise induces high expression of FGF23 resulting in an increased exercise endurance [148]. In addition, exogenous treatment of FGF23 contributed to reduced exercise-induced ROS and H_2_O_2_ production, increased PPARδ and citrate synthase activity, thus, enhancing mitochondrial function in skeletal muscle [148]. However, the new relationship between FGF23, mitochondrial functions, and skeletal muscle is not well understood and warrants more research to understand the molecular mechanisms of FGF23 regulation.

### 2.6. Clinical Significance of FGF23

It is a well-known fact that diseases characterized by excessive blood concentrations of intact FGF23 lead to renal phosphate wasting and low-circulating 1,25(OH)2D3 levels in patients with functional kidney [150]. In humans, the expression of FGF23 is modified in several diseases. Elevated levels of FGF23 were reported mainly in genetic X-linked hypophosphatemia (XLH) and non-genetic tumor-induced osteomalacia (TIO), a rare condition of hypophosphatemia caused due to increased levels of FGF23 and associated with low levels of 1,25-(OH)2D [151]. Elevated levels of FGF23 have been quantified in a majority of XLH patients and hypophosphatemia mice, an orthologous animal model of XLH [152]. Furthermore, elevated levels of FGF23 are also associated with CKD-mineral bone disorder [153]. CKD is an extremely complex disease with complications like renal failure and bone mineral disorder. It has been observed that elevated levels of FGF23 were correlated with bone mineral density in the lumbar spine site in CKD patients [154]. Thus, FGF23 can be used to predict bone loss at the lumbar spine site in the end stages of CKD [154]. On the other hand, a low level of FGF23 is reported in hyperphosphatemic tumoral calcinosis [155]. 

Mutation in FGF23 is associated with ADHR through a gain of function mutation(s) in the *FGF23* gene [56]. This mutation affects residue at positions 176 and 179, often called the RXXR site, by the subtilisin-like pro-protein convertases (SPC), which prevents proteolytic cleavage of FGF23 [56]. Later, a novel missense mutation was identified in autosomal recessive hyperphosphatemic familial tumoral calcinosis patients and the level of FGF23 was found to be lower [156]. 

FGF23 has been identified as a mediator of left-ventricular hypertrophy [157,158]. Angiotensin II (ATII) is also a strong trigger for the development of cardiac hypertrophy [159]. Interestingly, one study demonstrated that FGF23 enhanced the intracellular expression of ATII from cardiomyocytes in an autocrine manner [159]. The study suggested that the ATII’s FGF23-induced enhanced synthesis may work through the secretory pathway that involved angiotensin-converting enzyme, but the exact mechanism of this relationship is still unknown [159]. However, the increased expression of both ATII and FGF2-FGFR4 signaling induced the development of cardiac hypertrophy through IP3-nuclear Ca^2+^-dependent signaling [159]. Both in vivo and in vitro studies indicated that FGF23 promotes cardiac hypertrophy and fibrosis through the activation of the local renin–angiotensin–aldosterone system in the heart [160].

## 3. Comparison of Amino Acid Sequences of FGF2 with Endocrine FGFs

**Figure 3:**
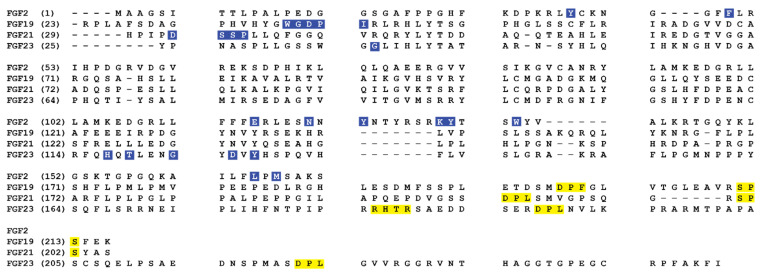
Multiple sequence alignments of human FGF2, FGF19, FGF21, and FGF23. The FGFR binding residues (blue) and klotho binding residues (yellow) are highlighted.

To further discuss the distinction of the structure and function of the endocrine FGF, multiple sequence alignment was performed and compared with a paracrine representative, FGF2 (Figure 3). Paracrine FGFs consists of 5 FGF subfamilies; FGF1, FGF4, FGF7, FGF8, and FGF9 [3]. These FGFs contain a signal sequence at the N-terminus and a heparin-binding site (HBS) at the C-terminus [3]. Functionally, the paracrine FGFs control a broad range of activities—developmental and physiological processes [7,10,11]. Itoh et al. (2016) showed that both endocrine and paracrine FGFs are important for maintaining proper health in mice and humans [161]. Paracrine FGFs exert their biological function by binding to FGFRs whereas endocrine FGFs mediate their biological responses by binding to klotho co-receptors with the FGFRs [5,6,7,8]. Due to these prominent functional variances, the investigation of endocrine FGFs moved into sequence comparisons between the endocrine subfamily and FGF2 to determine the molecular differences that may explain such drastic dissimilarity between endocrine and paracrine modes of action and subsequent downstream physiological activities.

Figure 3 shows the structural comparison of the endocrine FGF subfamily with a paracrine FGF, FGF2 [6,162,163,164,165,166]. Multiple sequence alignments of FGF2 with endocrine FGFs display that the primary sequence of the β10–β12 heparin-binding segment differs across the FGF2 and FGF19, 21, and 23 [167]. Most of the sequence divergence between FGF19 subfamily members stem from the HBS regions, namely the β1–β2 loop and the segment between the β10 and β12 strands of these ligands. The identity between the HBS of endocrine subfamily members is at best 13%. Exclusion of the HBS from the alignment improves the sequence identity between FGF19 subfamily members to above 40% [167]. In paracrine FGFs, however, the degree of sequence divergence at the HBS region is much less and is comparable with the degree of divergence in other regions of the β-trefoil core.

All the paracrine FGF subfamilies (FGF1, FGF4, FGF7, FGF8, and FGF9 subfamilies) contain a definite GXXXXGXX(T/S) motif in the heparin-binding loop (Figure 3). The GXXXXGXX(T/S) motif, also known as the glycine box, delivers a common conformation to the heparin-binding segment (β10–β12 strands) [168]. In this motif, first and second glycine residues make hydrogen bonds with a conserved glycine amino acid in β3 and β7 strands. These hydrogen bonds are important for the configuration of the β11 strand [167]. The hydroxyl group of the terminus threonine/serine amino acid forges a hydrogen bond with the backbone of the second glycine in the glycine box. The crystal structure of heparin/SOS bound FGF2 has shown that backbone and side-chain atoms of FGF2 form hydrogen bonds with the sulfate and sugar backbone atoms of heparin sulfate [169]. This hydrogen bonding provides the binding energy necessary for the interaction of heparin with FGF2.

Compared to the paracrine FGFs, the FGF19 subfamily displays the least sequence identity amongst its members (FGF19, FGF21, and FGF23) [6,10,170,171,172] (Figure 3). The pairwise sequence identity between the core regions of members of paracrine FGF subfamilies ranges from 88% for FGF9 and FGF16 to 54% for FGF7 and FGF10 [173]. Contrastingly, the identity between the core regions of members of the endocrine subfamily ranges between 33% for FGF21 and FGF23 and 38% for FGF19 and FGF21. Major sequence variance between FGF19, FGF21, and FGF23 arises from the heparin-binding region, mostly the β1–β2 loop and the segment between the β10 and β12 strands of these endocrine FGFs [167] (Figure 3).

The glycine box sequence (GXXXXGXX(T/S)) is missing in the endocrine FGFs and the heparin-binding loop is comparatively shorter than the one observed in the paracrine FGFs (Figure 3); inferring that the heparin-binding region of FGF19, FGF21, and FGF23 adopt different conformation than that observed in paracrine FGFs [167]. This different conformation disrupts the hydrogen bonding between heparin sulfate and amino acid residues present in the heparin-binding site. Hence, the endocrine FGFs bind weakly to heparin sulfate and forms a stronger interaction with a co-receptor, klotho. Therefore, this subfamily acts in an endocrine manner rather than as a paracrine.

## 4. Structural Features of Paracrine and Endocrine FGFs

Herein, the crystal structures of each endocrine FGFs are discussed to provide key information regarding their structure-function relationships. Furthermore, the reason behind the characteristic low affinity for heparin that facilitates the endocrine FGFs to act as hormones may be provided by contrasting the structures of endocrine FGFs’ to a representative of paracrine FGFs, such as FGF2 [3,7,8,15].

### 4.1. Structural Features of Paracrine Secretions 

**Figure 4:**
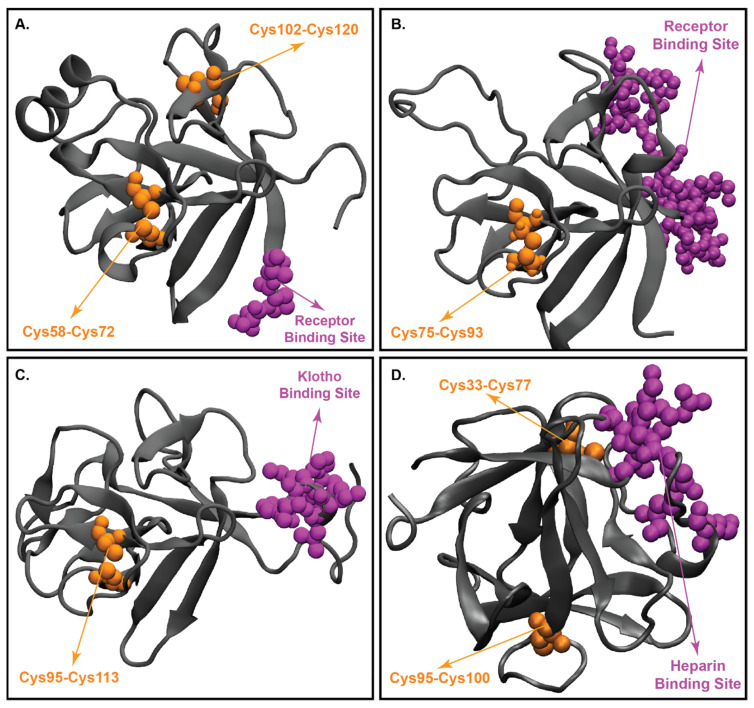
Crystal structures of endocrine FGFs. (Panel-**A**): Crystal structure of FGF19. (Panel-**B**): the crystal structure of FGF21. (Panel-**C**): the crystal structure of FGF23. (Panel-**D**): the crystal structure of paracrine FGF2. The protein backbone is represented as a grey cartoon, Disulfide bonds are shown as yellow spheres and receptor binding sequence is represented in magenta; All the crystal structures were prepared using visual molecular dynamics (VMD) software [176].

FGF1 and FGF2 are prototype members of the fibroblast growth factor family. Initially, FGF2 was found to be a 146 amino acid long protein isolated from the pituitary gland [2]. Like all the paracrine FGFs, FGF2 binds strongly to glycosaminoglycans such as heparin sulfate, which in turn helps ease the purification of FGF2 using heparin-Sepharose affinity chromatography [174]. Just like other members of the FGF family, FGF2 shares the common “β-trefoil fold” [175] (Figure 4D). Out of 146 amino acids, 26 residues are conserved in all members of the FGF family. Of these 26, 7 are glycine/proline (G/P) and 13 are hydrophobic amino acids. The conserved G/P forms the turns between the β-strands, thereby playing a major role in the structural scaffold. FGF2 has 4 cysteine residues (Cys 33, 77, 95, and 100). Of these four cysteines, Cys33 and 100 are conserved amongst the FGF family members [2].

As discussed in the previous section, FGF2 is a heparin-binding protein. The binding of FGF2 to heparin induces dimerization of FGF, which in turn, is a prerequisite for FGFR activation and signaling [177]. FGF2 has two different receptor binding sites namely, primary and secondary. These binding sites were identified based on site-directed mutagenesis studies. The primary binding site is conserved in the FGF family and provides a significant binding interaction with FGFR. This primary binding site comprises of Y24, E96, N101, Y103, L140, and M142 [165,166]. Alternatively, the secondary receptor binding site consists of K110, Y111, and W114 [178,179,180,181]. 

### 4.2. Structural Characteristics of FGF19

A conservative structural similarity exists between FGF19 and other FGF family members. Like other FGFs, FGF19 forms a β-trefoil like structure (Figure 4A). β strands 1, 4, 5, 8, 9, and 12 are involved in the formation of β-trefoil with a well-formed β barrel at the base of the trefoil. Extended β-sheets of strands 1 and 9 form β-hairpin-like structures [13]. One of the special features that separate FGF19 from other FGFs is the presence of two disulfide bonds. These two disulfide bonds are situated to stabilize β hairpins formed by the β sheets of strand-1 and -9. The first disulfide bond is located between -C58 of β-strand 2 and C70 of β-strand 3, and the second is situated between C102 of β-strand 6 and C120 of a loop between strands-7 and -8. C120, in the case of FGF19, is conserved across the FGF family [4]. The first disulfide bond in FGF19 aids in holding the extended loop formed by strands-1 and -2. The second disulfide bond stabilizes the protein backbone. Correlation of the extended loop between strands-7 and -8 by only one residue and inclusion of disulfide link leads to correct folding of FGF19, thereby enhancing the stability of the protein [13]. 

One structural difference between FGF19 and other FGFs is the presence of an extended N-terminus [13]. The presence of this extended loop is also observed in FGF7 and FGF10 for providing a key interaction with the “b” splice form of FGFR2 [182], but there is no evidence to suggest a functional role for extended β1 in FGF19. FGF19 also shows an extended loop of eight amino acids linking strands β1 and β2 [4]. This conformation is stabilized by the heparin with its sulfate ions forming hydrogen bonds with the amide groups of FGF19 (H53, L55, and S56). Additionally, the extended loop interaction with the sulfate group of heparin, makes it functionally relevant as this is spatially closer to the heparin-binding domain of FGF19 [13]. 

Another most important structural feature revolves around strand β11 of FGF19 [13] (Figure 4A). Unlike other FGFs, this strand is missing a loop observed in the heparin-binding FGFs [179,183] (Figure 4A). It has been demonstrated that β11 is present in most of the FGF subfamily members and is responsible for heparin-binding [182,183] (Figure 4D). Therefore, the disordered loop in β11 of FGF19 explains the reduced heparin-binding affinity. This conformational change (disorientation) brings great significance to the discussion related to the requirement of heparin-binding for FGF downstream signaling [184]. In particular, the disorientation allows FGF19 to have a low affinity towards heparin thus, permitting the protein to diffuse into the blood and act as a hormone, while evading its paracrine/autocrine functions [7,15,184]. Interestingly, according to Wu et al. (2007), FGF19 shows increased FGFR4 activation with increasing heparin concentration [185]. However, heparin-binding experimental data suggest FGF19 requires lower concentrations of NaCl (less than 400 mM) to release from heparin-bound column compared to the FGF1 subfamily members (1.5 M). FGF1 and FGF2 form strong interactions with heparin through the backbone and sulfate groups around the disoriented portion of FGF19. This renders FGF19 sterically unconducive to bind to heparin in the same manner as FGF1 and FGF2 [13]. 

### 4.3. Three-Dimensional Structure of FGF21

Like other FGFs, FGF21 is a 181 amino-acid protein that functions in an endocrine fashion and requires KLB, a single transmembrane glycoprotein to bind to FGFR [53]. FGF21 has a 13-residue N-terminus and a 40-residue C-terminus with a β-trefoil core domain (FGF21 core) [44,53] (Figure 4B). However, unlike other members of the endocrine FGF subfamily (FGF19 and 23), there is only limited information regarding the structure of FGF21. Most recently, the core structure of FGF21 was elucidated by nuclear magnetic resonance spectroscopy (Figure 4B), and the crystal structure of the C-terminus domain—KLB binary structure was determined at high resolution [53,186]. 

The core sequence of FGF21 was thought to have a typical 120 amino acid β-trefoil core structure like many FGFs [43]. However, it has been recently indicated that the core region of FGF21 only has 11 β-strands (1–10, 12) and misses the canonical β-11 strand. β10 is linked to β12 through a proline-rich 18-residue disordered random coil [53]. Such replacement leads to divergence in topology and structures of FGF21 compared to typical FGFs and even other endocrine FGFs, FGF19 and 23. The β10-β12 region of FGF21 is shorter than that of FGF19 and FGF23, with little to no sequence identity shared amongst the three proteins [187]. Additionally, the overall sequence identity increases to 40–45% if this region is omitted during multiple sequence alignment of all endocrine FGFs, indicating it to be the key region accounting for the low sequence identity amongst the members of endocrine FGFs [187]. Another region of endocrine FGFs called the HBS region (β1-β2 loop), also shares little sequence identity and structure amongst its members [167]. If the HBS region was omitted, the alignment identity of the members will increase to 40% [167]. Furthermore, additional data reveals that the β2-β3 hairpin structures and neighboring segments also display high flexibility, which decreases the stability of the protein [53].

The N-terminus and C-terminus of FGF21 play different roles when the protein is bound to FGFR and KLB [43]. Specifically, the C-terminus has been shown to constitute the binding site for KLB and the N-terminus domain plays a crucial role in receptor activation [43,44]. Serial truncations of the C-terminus domain of FGF21 weakened the ability of the protein to interact and bind to KLB [43,44]. In contrast, when the N-terminus was subjected to the same serial truncations, it did not affect the ability of FGF21 to bind to KLB [43,44]. Instead, this truncation affected the efficacy of FGFR activation by FGF21 [43,44]. It is reported that residues 198–200 of FGF21 C-terminus (FGF21CT) are flexible and can be potentially accessible for proteolysis to abolish the binding of FGF21 to the extracellular binding sites in human KLB (sKLB) [186]. Furthermore, FAP (introduced in Section 2.4) is believed to be responsible for cleaving FGF21 at proline 171 and inactivating the protein, thus providing the mechanism for termination of FGF21 signaling [123,188]. Interestingly, it is also believed that FGF21 signaling occurs solely through FGFR and not through the co-receptor, KLB, because KLB only has a few amino acid residues in its intracellular domain [43]. Therefore, the N-terminus of FGF21 is seen as a point of interest to investigate the efficacy of FGFR activation of FGF21 [43].

### 4.4. Structure of FGF23

FGF23 is a 32 kDa protein produced in the bone by osteoblasts and osteocytes under physiological conditions. Its co-receptor, KLA, is mainly synthesized in the distal kidney tubules, parathyroid gland, and choroid plexus [189,190]. These tissues are, therefore, the targets for FGF23 [191,192]. KLA binds to the C-terminus of FGF23 and FGFR to accelerate the ligand-receptor affinity [187]. The protein is produced in the bone and is involved mainly in the mineral metabolism of phosphate and vitamin D [193]. FGF23 decreases the reabsorption of phosphate from urine and the synthesis of 1,25(OH)2D3, vitamin D [193]. The low affinity of FGF23 to heparin allows it to enter the circulation and function as an endocrine secretion [194].

Like other endocrine FGFs, FGF23 has an atypical β-trefoil fold and does not have a β11 strand [187] (Figure 4C). β10 and β12 conformation of FGF23 is different from the β10 and β12 conformation of the paracrine FGFs [187]. Specifically, FGF23 lacks the β11 strand in its core structure, a consistent characteristic shared amongst FGF19, 21, and 23 [187]. Such divergence explains the non-canonical β-trefoil core structure of FGF23 [187]. Additionally, FGF23 and its endocrine FGF cousins also have a shorter β9-10 loop, compared to paracrine FGFs, and may influence the conformation of the β10-12 loop [187]. Despite the seemingly uniting structure features amongst endocrine FGFs, there exist distinct differences between them, especially in the β1-2 region and HBS region, as described previously [187].

Another difference amongst the endocrine subfamily members is the C-terminus sequence. The C-terminus of FGF23 differs from those of FGF19 and FGF21 in possessing 89 amino acids that form two parts, called R1 and R2 [69]. Both parts bind to KLA with high affinity, while FGF19 and FGF21 bind to KLB through the same binding site [69]. In mammals, the R2 is flanked by two cysteine residues Cys206 and Cys244. The disulfide bridging of two cysteine does not influence the susceptibility of FGF23 proteolytic digestion. Also, R2 in its oxidized form can form an active ternary complex with KLA and FGFR1c [69]. Interestingly, the 73-residue C-terminus of FGF23 is suggested to be innately flexible that causes difficulties in the crystallization process of FGF23 [187].

Many important structural and physiological significances of FGF23 involve its C-terminus. For example, circulating levels of physiologically active FGF23 are regulated by a highly conserved proteolytic cleavage site at the C-terminus to produce two forms of FGF23 in the bloodstream [56,195,196,197]. The first form is the full-length form called wild-type FGF23 that contains Tyr25-Ile252 and is physiologically active. The second form is the truncated form consisting of residues only from Tyr25-Arg179 called core FGF23 that does not have the 73 amino acids of the C-terminus, thus cannot assert its activities through the C-terminus [56,195,196,197]. This conserved cleavage site within FGF23 is a furin proconvertase recognition site and it ranges from Arg176 to Arg179, often called the RXXR (RHTR^179^) site [56,197,198]. This cleavage is processed by SPC and cleaves between arginine 179 and serine 180, which leads to the inactivation of FGF23 [199]. As mentioned previously, this cleavage site is often mutated in ADHR patients [56,197,198]. The mutations usually replace arginine residues with glutamine at either position 176 or 179 [56,197]. Another mutation was also found at position 179, where arginine is replaced by tryptophan [56,197]. The mutations result in an FGF23 mutant with resistance to proteolysis and an increased half-life, leading to elevated levels of 1,25(OH)2D3, and decreased levels of serum phosphorous [56,197]. Both phenotypes are characteristics of ADHR condition [56,197]. Thus, the cleaving process is essential in the normal regulation of phosphate homeostasis [200].

FGF23 undergoes multiple post-translational modifications that influence the stability and susceptibility of FGF23 against proteolysis. For example, to protect FGF23 from C-terminus cleavage, the protein is O-glycosylated by GalNAc-transferase at the C-terminus RXXR cleavage site [199,200]. Specifically, within the RXXR cleavage site, there is a threonine residue at position 178 that is shown to be an O-glycosylated position for FGF23 [200]. Additionally, FGF23 undergoes modification through disulfide bond formation (C95–C113) and O-glycosylation by uridine diphosphate-N-acetyl-α-D-galactosamine: polypeptide N-acetyl galactosaminyltransferase 3 at multiple sites to produce the mature 32 kDa protein [201]. Another modification of FGF23 is its phosphorylation at the region of the cleavage site [202]. The serine residue at position 180 plays a dual role: (1) a phosphorylation site for FGF23 and (2) a cleavage site for SPC [56,202]. This phosphorylation process is conducted by a kinase family called: family with sequence similarity 20, member C (Fam20C), which phosphorylates proteins at Ser-X-Glu motifs [202]. Fam20C phosphorylation is believed to prevent the glycosylation at the cleavage site as well as expose the site for the subsequent proteolysis process [202]. Combining with the other modification processes, FGF23 shows to have a dynamic and complex regulatory system that warrants further investigations.

## 5. Klotho Proteins

Unlike paracrine or autocrine FGFs, members of the endocrine sub-family lack the classical heparin-binding pocket, which is usually conserved within paracrine FGFs’ core region [167]. Additionally, another region, the β10-β12 hairpin region of endocrine FGFs, also shows a distinct difference when compared to the paracrine FGFs. This region in FGF19, 21, and 23 lack the GXXXXGXX(T/S) motif that is present throughout the paracrine FGF subfamilies [167]. The absence of this motif is believed to be the reason for the conformation of the β10-β12 regions of endocrine FGFs [187] and may contribute to the low heparan/heparin-sulfate binding affinity [53]. Hence, they require the *klotho* gene family as co-receptors to bind to the FGF receptors. Initially, the *klotho* gene was identified as a mutated gene in mice which developed premature aging [53]. Several studies have reported that overexpression of klotho in mice can result in a longer lifespan, whereas defect in *klotho* gene expression was found to lead to premature aging [203], including short lifespan [204] and muscle atrophy [205]. These results suggest that the *klotho* gene may aid in the regulation of age-related diseases.

Based on the primary sequences, the klotho family consists of α-, β-, and γ-klotho [186,206,207,208]. They are all single-pass transmembrane proteins [205]. The extracellular domain of the klotho protein has two homologous domains, KL1 and KL2 [203]. These two homologous regions share sequence similarities with β- glucosidase enzyme present in plants and bacteria [187]. However, the klotho protein does not exhibit any β- glucosidase-like activity [167,187,203,205,209].

**Figure 5:**
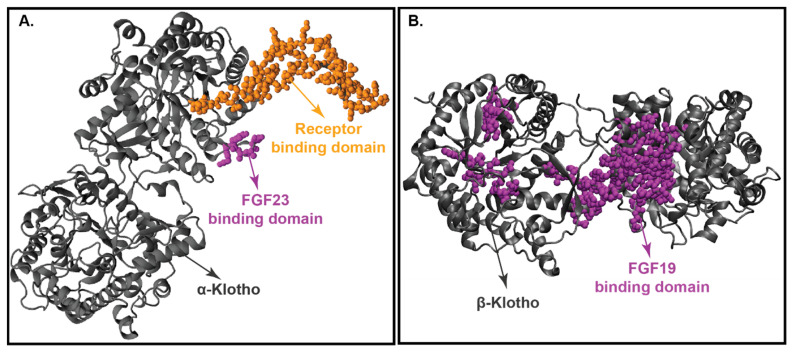
Crystal structure of klotho proteins. (Panel-**A**): Crystal structure of KLA. (Panel-**B**): Crystal structure of KLB. Both KLA and KLB’s backbone is represented in grey, protein binding domain (FGF23 for KLA and FGF19 for KLB) shown in magenta, and receptor binding domain is represented in yellow.

The crystal structures of KLA with FGF23 are depicted in Figure 5A [6,172]. This crystal structure supports the notion that FGF23 binds to FGFRs (FGFR 1c, 3c, and 4) in the presence of a co-receptor, KLA [68,210]. Furthermore, the KLA crystal structure shows that there are 2 extracellular domains, KL1 (E34 to F506) and KL2 (L515 to S950), in KLA [211]. The N-terminus of FGF23 binds to the KL2 domain of KLA, which in turn interacts with the D1 receptor of FGFR, and the C-terminus of FGF23 forms an active ternary receptor complex by binding to a pocket created by the KL1 and KL2 domains [210,212]. In mammals, KLA goes through a proteolytic cleavage to create soluble KL1 and KL2 protein fragments. Several reports reveal that the soluble KL1 and KL2 fragments lead to KLA-independent signaling [210,212].

The crystal structure of KLA reveals that it binds tightly to FGF23 and FGFR1c [68,69]. In this binding process, KLA increases the proximity of FGF23 and the receptor (FGFR1c), thereby increasing the binding affinity of FGF23 with the receptor FGFR1c [68,69]. In this complex, FGF23: FGFR1c: KLA, KLA exists in an extended conformation adopting a triosephosphate isomerase (TIM) structural topology [213]. Both the KL domains are connected by a proline linker (P507–P514). KL1 and KL2 interact with each other via N-terminus preceding the β1 strand and the α7 helix of KL1, and the β5α5 and β6α6 loops and α7 helix of KL2 [213]. These connections stabilize the structure of KLA and create a deep cleft between the KL1 and KL2 domains [213]. This deep cleft along with a central β- barrel cavity serves as a binding pocket for FGF23. On the other hand, the β1α1 loop for KL2 projects outwards from the KL2 core and attaches itself to the FGFR1c domain [214].

Figure 5B displays the crystal structure of KLB with FGF19. Similar to KLA, KLB has 2 extracellular ligand-binding domains, KL1 and KL2. Shi et al. (2018) performed the differential protection factor plot and determined the amino acids that are potentially involved in the binding of KLB to FGF21 [207]. The residues involved are 316–328 (α5 helix), 375–395 (β5-α6 loop), 416–422 (β7 strand and β7-α7 loop), 430–438 (α7 helix), 523–531 (β9 strand), 688–697 (β11 strand and β11-α11 loop), 746–759 (β12-α12 loop) and the membrane-proximal region of the KL2 domain [215].

Comparison of the crystal structure of FGF19 and FGF21 bound KLB infers that FGF19 and FGF21 occupy similar binding sites on KLB. Multiple sequence alignment of the C-terminus of FGF19 and FGF21 discloses two signature motifs—the D-P-L/F motif and the S-P-S motif [208]. The D-P-L/F motif is important in maintaining the intramolecular hydrogen bonding interactions in KL1 whereas the S-P-S motif aids in the identification of the pseudo-substrate region in KL2 [216]. These signature motifs are conserved among various species in both FGF19 and FGF21, thereby implying that FGF19 and FGF21 exhibit the same molecular interactions with KLB [13,74,185].

## 6. Interaction of Endocrine FGFs with FGFR1c and Klotho Proteins

It is well-documented that the endocrine FGFs are involved in many metabolic functions and diseases (ADHR, XLH, obesity, T2DM, etc.) through their interactions, or lack thereof, with the co-receptors KLA/KLB and FGFRs [15,55,56]. Therefore, to gain a better understanding of these physiological functions of endocrine FGFs and their roles in human conditions, the interaction mechanism of endocrine FGFs to their respective receptors and co-receptors will be highlighted in the ensuing sections below.

### 6.1. FGF19/21-Receptor Interaction

Paracrine FGFs interact and activate FGFRs through a high-affinity interaction with heparan sulfate proteoglycan (HSPG) [53,217] (Figure 4D). Such appearance of HSPG with high affinity to FGFs helps induce notable and prolonged FGFR dimerization [218]. On the other hand, the endocrine subfamily members have a weak binding affinity to HSPG [43,44,46,53]. This low affinity to HSPG is believed to be caused by aberrant conformation with the entailed steric hindrance of the β10–12 regions (Figure 4A–C) as indicated previously [53]. However, it is established that FGF21 requires KLB to interact with its cognate receptor [187,206,219] to the aforementioned list of FGF21-binding receptors: FGFR1c, 2c, 3c, and 4 [217,220].

**Figure 6:**
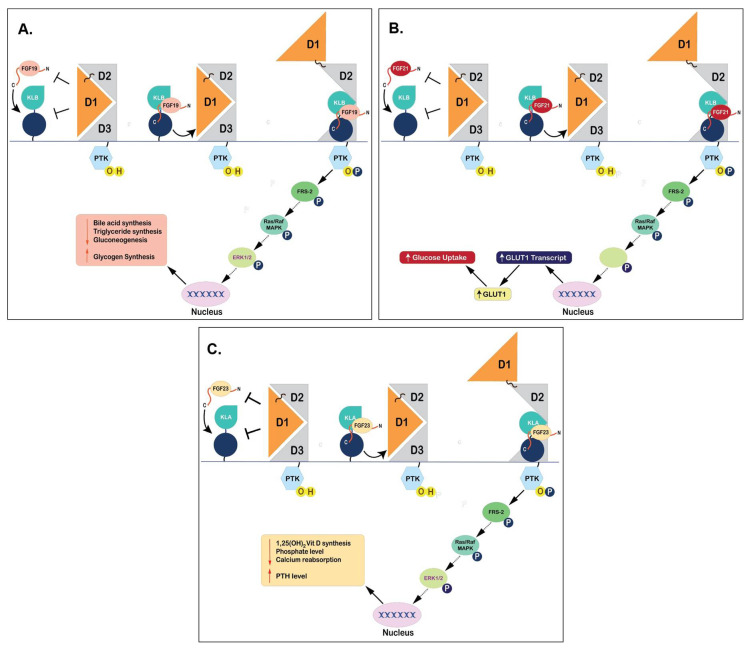
Binding Mechanisms of Endocrine FGFs to FGFR1c and klotho proteins. (Panel-**A**): Binding of FGF19 to KLB and FGFR1c that results in subsequent repression of bile acid synthesis. (Panel-**B**): Interaction of FGF21 to its receptors (KLB and FGFR1c) and subsequent activation of downstream signaling pathways leading to physiological effects of glucose uptake in adipose tissues. (Panel-**C**): Schematic representation of binding of FGF23 to KLA and FGFR1c and induction of downstream signaling pathways to regulate phosphate levels.

The interactions between FGF21 and KLB through FGF21 C-terminus is well-documented [217]. The structure of FGF21CT and sKLB complex revealed that FGF21CT binds to the interface between two domains D1 and D2 of the sKLB [186] (Figure 6B). On these two domains of sKLB, there are two binding sites for FGF21CT, called sites 1 and 2, that are 30 Å apart [186]. Site 1 is on the domain D1 and binds to FGF21CT in the segment, P186-V197, through hydrophobic interactions with several β-turns [186]. Site 2 is on domain D2 and binds to FGF21CT in the segment of S200-S209, which contains a high amount of hydroxyl side chain [186]. Interestingly, this second binding site mimics the binding interactions between glycoside hydrolase 1 enzyme and its substrate. Furthermore, the S204-P205-S206 sugar-mimicking motif at the end of FGF21CT interacts strongly with the “catalytic” E693 of sKLB and resembles the substrate interactions in GH1 [186]. Combining these aspects, the data suggest that FGF21 may be mimicking a glycosidic substrate [186].

The S-P-S sequence motif is also present at the C-terminus of FGF19, specifically S211-P212-S213, which helps in binding to KLB (Figure 4A and Figure 6A) [186]. FGF19 and 21 present similar mechanistic interactions while interacting with KLB (Figure 6A,B), which is different from FGF23 that is angled towards KLA (Figure 6C) [186]. This is because FGF23 does not contain a similar sequence motif, making it unable to bind to KLB but binds to KLA through different mechanistic pathways [186]. Another important motif called the D-P-F/L sequence, D198-P199-F200 specifically, is also involved in the interaction with klotho through hydrogen bonding to maintain the complex conformation of FGF19 and KLB [186]. This D-P-F/L motif is also conserved in FGF21 and 23, specifically D192-P193-L194 in FGF21 and D188-P189-L190 in FGF23, contributing to the same function of binding to their respective klotho proteins [69,186] (Figure 4C and Figure 6C).

The N-terminus is not required for KLB binding, but it does contribute towards the binding and activation of FGF21 to FGFR [43,44]. FGFR1c is shown to be a preferred receptor for binding to FGF21 [217]. Removal of 5 amino acids from the N-terminal end of FGF21 appears to reduce responses in signaling and functional activity [44]. As mentioned previously, FGFRs contain 3 immunoglobin (Ig)-like domains (D1-D3) and a linker between D1 and D2 domains [217]. Interestingly, the autoinhibitory D1/linker region of FGFR1c, which comprises of D1 domain and D1-D2 linker, can suppress the FGF21/FGFR1c interaction and FGFR1c//KLB interaction, rendering FGF21 unable to stimulate signaling with FGFR1c alone without KLB [217].

FGF21 is suggested to interact with D2-D3 extracellular regions of FGFR1c directly through its N-terminus [217](Figure 6B). Furthermore, the β1-β2 loop in FGF21 is important for the FGFR interaction and activation as it is the direct contact to the D2 domain of FGFR1c [53]. However, this β1-β2 loop can be disturbed by the destruction of another loop, the β2-β3 loop, thus affecting the binding of FGF21 to the D2 domain of FGFR1c [53]. The β2-β3 region of FGF21 is also quite important due to its role in providing stability to the triangular array architecture of FGF21. This region is also flexible, thus making FGF21 unfavorable for the interaction with FGFR, allowing FGF21 to escape to the local extracellular matrix [53]. Although the direct interaction of FGF21 and FGFR1c without KLB is weak and transient, it is thought to be similar to paracrine FGFs-FGFRs in a 2:2 ratio complex, with multiple points of interactions [169,217].

### 6.2. FGF23 Forming Complex with FGFR1c and KLA

FGF23 binds and activates FGF receptor tyrosine kinases (FGFRs) like FGFR1c, FGFR3c, and FGFR4 to produce downstream signaling and subsequently, regulates phosphate and vitamin D levels [187,191,192,221]. There is a preference for FGF23 to bind to the c isoform of FGFR1–3 and FGFR4. This preference was known to be originated during the interaction studies between FGF23 and FGFR1c receptors. These studies showed that the signaling of FGF23 was negatively affected when the specific serine residue in the FGFR1 c isoform was replaced by a tyrosine residue to convert it to the b isoform of the receptor [214]. FGF23 was found to bind to FGFR1c between the D2 and D3 domains. However, FGF23 makes weaker contacts with D3 and D2-D3 linkers when compared with the paracrine FGFs [214].

Another important difference between the interaction of FGF23 with its receptor and paracrine FGFs with their receptors is the presence of co-receptor KLA [222]. Studies have shown that KLA enhanced the binding affinity of FGF23 to FGFR by a factor of 20, further emphasizing the role of KLA in the successful binding and activation of FGF23 to FGFR [222]. The R1 and R2 sections of the C-terminus of FGF23 play an important role in binding FGF23 to its co-receptor KLA [69,214]. This is because both R1 and R2 have D-P-L/F motifs and specific Asp188-Pro-Leu-Asn-Val-Leu193 residues in the C-terminus of FGF23 form a compact, rigid cage-like conformation when bound to its co-receptor KLA [69,214]. These 2 parts with similar D-P-L/F motifs indicate that one molecule of FGF23 might have two binding sites for KLA [69]. Variants of FGF23, containing either R1 or R2 repeat, show similar binding affinities to KLA when compared to the native FGF23 [69,186], thereby permitting the use of either FGF23 variants as antagonists in studies related to the functions of FGF23 in vitro and in vivo [69,186]. Interestingly, this D-P-L/F motif is conserved amongst endocrine FGFs to serve as interaction points for klotho proteins [69,186]. Although all vertebrate FGF23 proteins have long C termini, only the mammalian FGF23 proteins have a second repeat (R2) that is homologous to the klotho binding regions of FGF19, 21, and 23 [69].

**Figure 7:**
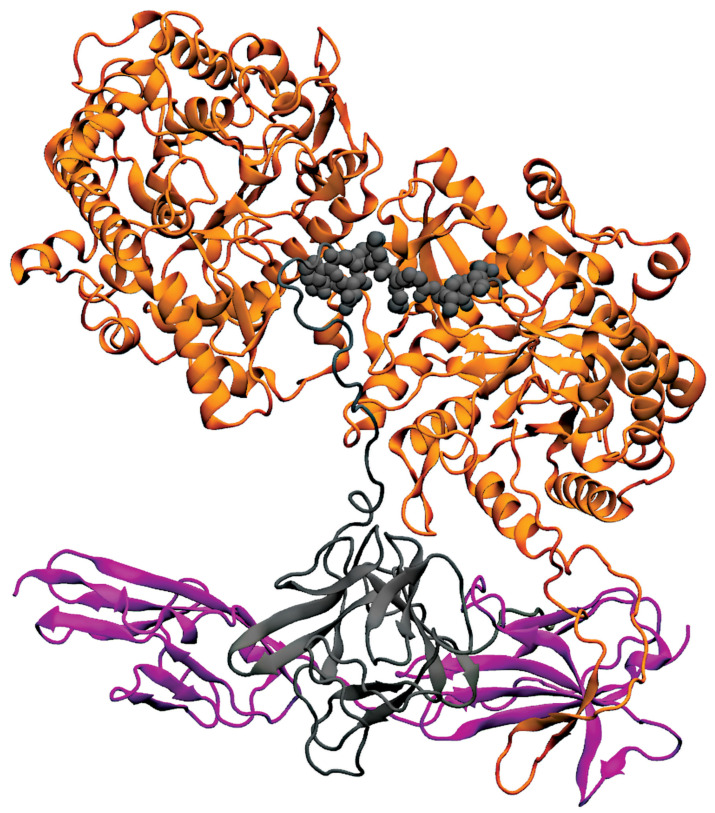
Crystal structure of Protein receptor (FGF23-FGFRc1-KLA) complex. Both KL1 and KL2 domains of KLA backbone are represented in orange, FGF23 protein shown in grey, and receptor FGFR1 is represented in magenta.

The crystal structure of the ternary complex of FGF23, FGFR1c ectodomain (comprises of domains D2, D3, and D2-D3 linker) and KLA, shows that KLA contains two domains, called KL1 and 2, which are connected by a short proline-rich stiff linker (Pro-507 to Pro-514) (Figure 6C). KL1 is on top of KL2, held by a few interdomain contacts between KL1 (the N-terminus preceding the β1 strand and the α7 helix) and KL2 (the β5α5, β6α6 loops, and the α7 helix) [214]. These interdomain interactions create a deep cleft between the 2 KL domains and merge with the β-barrel structure in KL2 to form a large pocket [214]. This large pocket is the binding site for the C-terminus tail of FGF23 past the protein’s RXXR proteolytic cleavage site [214] (Figure 7). At the same time, the long β1α1 loop of KL2 clamps on FGFR1c’s D3 domain and assists in connecting FGFR1c to KLA [214]. Due to this interaction, this part of KL2 is called the receptor binding arm (RBA) [214]. Both soluble and transmembrane KLAs have the same function as the co-receptors for FGF23 signaling [214]. However, the soluble form of KLA that does not have the binding arm (RBA), fails to form a binary complex with FGFR1c ectodomain in solution and hence could not support FGF23 signaling [214,223].

## 7. Applications and Clinical Trials Involving Endocrine FGFs

**Figure 8:**
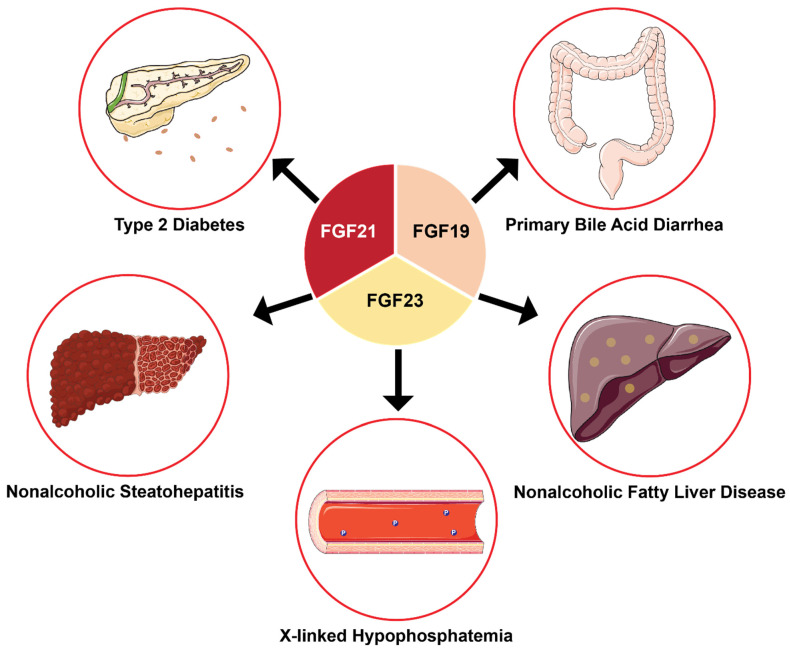
Biomedical applications of endocrine FGFs that target specific human diseases and conditions. Current biomedical and clinical applications of endocrine FGFs focus on treating various diseases. Many of these applications have advanced to Phase I or II of clinical trials and show optimistic results.

The roles of endocrine FGFs in mammalian species, especially human, physiology are undoubtedly important [49,141,161,204,224]. Likewise, their roles in human diseases and conditions, such as XLH, ADHR, T2DM, NASH to name a few are equally critical (Figure 8) [4,122,161,167]. Fortunately, the fundamental knowledge of endocrine FGFs is well-developed: from understanding the primary physiological roles of endocrine [5,49,80,141,204], to their individual structures and mechanism [44,69,185,204,209,224], and their physical interactions with other proteins [217,220,225]. Therefore, the trend of endocrine FGFs research is focused on applying the fundamental knowledge to biomedical and clinical applications where many of them are at various phases of clinical trials (Table 1) [6,9,99,168].

FGF19 and FXR affect the bile acid regulation and have been known to treat primary bile acid diarrhea [13,14]. FXR agonists were further developed based on the relationship of FGF19 with FXR [226]. This agonist reduces the expression of FGF19 in the liver, thus, decreasing hepatic bile acid secretion for treating bile acid diarrhea [226]. In a very recent clinical trial, Tropifexor (LJN452) a highly potent non-bile acid FXR agonist, was employed to assess the efficacy in a patient with primary acid bile diarrhea. Tropifexor at 60 μg once-daily dose for 14 days had an acceptable safety and tolerability profile in those patients [226].

Obeticholic acid (OCA, also known as 6a-ethyl-CDCA and INT–747) is another potent FXR agonist that has been tried for clinical studies with positive outcomes. The efficacy of OCA in the treatment of NASH was investigated through a randomized and multicenter clinical trial for 72 weeks. The trial’s result stated that OCA improves the histological features of NASH [227]. A further phase 2 clinical trial result also indicated that OCA stimulates FGF19, which leads to a reduction in bile acid synthesis and is found to be a clinical benefit in the treatment of bile acid diarrhea patients [228] (Figure 8).

Besides FXR agonists, NGM282- also referred to as Aldafermin (introduced in Section 1.1), appears to be one of most promising endocrine FGF-based therapeutic agents that is currently in the clinical trials. Preclinical studies on NGM282 showed that the drug can impact many NASH-relevant pathways such as inhibition of lipogenesis, improvement of insulin sensitization, correction of mitochondrial dysfunction, and reduction of liver inflammation [229,230]. In an open-label study, NGM282 reduced the liver fat content, improved serum markers, and reduced nonalcoholic fatty liver disease activity score in NASH patients rapidly and significantly after 12 weeks of treatment [229]. A phase II clinical trial has analyzed the safety and efficacy of NGM282 for the treatment of NASH (Figure 8) [231]. Administration of 3 mg or 6 mg of NGM282 was well tolerated in patients and it reduced liver fat content as well as liver inflammation and fibrosis [231]. Subsequently, another clinical study showed that NGM282 could represent a potent strategy to replace bariatric procedures with equally effective, but less invasive, treatments for NASH [232].

Simultaneously, the development of FGF21-based therapeutic agents also saw progress through several potential pharmacological products. These agents are centered on the ability of FGF21 to increase glucose uptake and reduce body weight, which is essential for treating obesity and diabetes [46,233,234,235]. One of such agents is Pegbelfermin (BMS-986036), a polyethylene glycol-modified (PEGylated) recombinant human FGF21 analog. In a randomized double-blind placebo control phase 2 clinical trial with Pegbelfermin administration for 16 weeks, results demonstrated that the drug was generally well-tolerated which could significantly reduce hepatic fat fraction in patients with non-alcoholic steatohepatitis [51]. Recently, a phase 2 trial was conducted to assess the safety and tolerability of Pegbelfermin in patients with obesity and T2DM, who were at risk for developing NASH. The results of the trial suggested that Pegbelfermin was generally safe, and suitable in patients with obesity and T2DM [52] (Figure 8).

Similarly, another attempt to develop FGF21 as a potential drug application comes in the form of a long-lasting FGF21 analog, called PF-05231023 [236] (Table 1). This analog combines an antibody scaffold, CovX-2000, with a modified FGF21 molecule and has an extended half-life in vivo when compared to the native FGF21 [236]. Specifically, in preclinical studies in mice, PF-05231023 was found to reduce body weight, blood glucose, and lipid levels [237]. In phase 1 clinical trial, PF-05231023 was found to be an essential molecule in lowering triglyceride (TG) levels in obese hyper triglyceridemic adult subjects with or without T2DM [238].

FGF23 has also been a desired target for therapeutical developments due to its essential role in many human conditions, such as XLH [239,240] (Table 1). One example is KRN23, an anti-FGF23 antibody which is a recombinant human IgG1 monoclonal antibody that binds to FGF23 and blocks its biological activity [239]. Several clinical trials have been carried out to assess the efficacy of KRN23 with XLH patients (Figure 8) [239,241,242]. A phase 1 trial showed a positive effect of KR23 on serum phosphate (P_i_), and its efficacy and safety profile suggest the essentiality of KRN23 in treating patients suffering from XLH [239]. Similar positive outcomes such as significantly increased serum P_i_ levels and favorable safety profiles were also reported in subsequent Phase I/II trials. In addition, open-label clinical trials indicated that KRN23 is a potentially effective XLH treatment [241,242]. KRN23 then entered a phase 2 clinical trial under the name Burosumab and demonstrated improvement of renal tubular phosphate reabsorption and serum phosphorus levels in children with XLH [243]. Later on, another open-label phase 2 clinical trial revealed that XLH children who received Burosumab treatment indicated a favorable safety profile with mild to moderate adverse events, no instances of nephrocalcinosis nor abnormal chemistry panel results [244]. Burosumab treatment increased serum phosphorus, and improved rickets, and prevented early declines in growth in younger children with XLH [244]. A randomized, active-controlled, open-label, phase 3 trial was conducted to compare the efficacy of Burosumab with conventional therapy consisting of oral phosphate and active vitamin D. Burosumab treatment resulted in significantly greater clinical improvements in rickets severity and growth among children with XLH [245].

Within Table 1, active and ongoing clinical trials for endocrine FGFs-based therapeutic agents are also included. However, due to the lack of published records from these active trials, we are unable to discuss the results and the implications of the use of these therapeutic agents on human health. The future of research on endocrine FGFs can be expected to be exciting as several new applications of endocrine FGFs are being explored. The saga of endocrine FGFs continues to be mystical and interesting.

**Table 1:** Key examples of endocrine FGF analogs and antagonists in clinical trials.

Trial Identifier	Status	Disease/Condition	Intervention	Phase	Outcomes
FGF19					
NCT01943045	Completed	Diabetes mellitus	NGM282	Phase 2	An ideal strategy for the replacement of bariatric surgery with equally effective and less invasive treatment for NASH [232].
NCT01585025	Completed	Chronic diarrhea	Obeticholic acid	Phase 2	Obeticholic acid was beneficial in the treatment of bile acid diarrhea [228].
NCT02443116	Completed	NASH	NGM282 or (Aldafermin)	Phase 2	The administration of 3 mg or 6 mg of NGM282 was well tolerated in patients and it reduced liver fat content as well as liver inflammation and fibrosis [229].
NCT01265498	Completed	NAFLD, NASH	Obeticholic acid	Phase 2	Obeticholic acid improves the histological features of NASH [227].
NCT02713243	Completed	Primary bile acid diarrhea	LJN452	Phase 2	Tropifexor at a daily dose of 60 μg for 14 days had an acceptable safety and tolerability profile in patients [226].
NCT02834780	Active, not recruiting	Advanced HCC	H3B-657	Phase 1	Ongoing
NCT02508467	Active, not recruiting	HCC	Fisogatinib (BLU-554)	Phase 1	Ongoing
FGF21					
NCT02097277	Completed	T2DM	BMS-986036	Phase 2	Pegbelfermin is generally safe, well-tolerated, in patients with obesity and type 2 diabetes mellitus [52].
NCT01673178	Completed	T2DM	PF-05231023	Phase 1	PF-05231023 is essential in lowering TG in obese hypertriglyceridemic adult subjects with or without T2DM [238].
NCT02413372	Completed	NASH	BMS-986036	Phase 2	A 16 week well-tolerable dose of Pegbelfermin can reduce hepatic fat fraction in patient with NASH [51].
NCT04541186	Recruiting	Severe hypertriglyceridemia	BIO89-100D	Phase 2	Ongoing
NCT03486899	Active, not recruiting	Liver fibrosis, NAFLD, NASH	BMS-986036	Phase 2	Ongoing
NCT03486912	Active, not recruiting	Hepatic cirrhosis, Liver fibrosis, NAFLD, NASH	BMS-986036	Phase 2	Ongoing
FGF23					
NCT00830674	Completed	XLH	KRN23	Phase 1	A favorable safety profile with Burosumab increased serum phosphorus levels, improved rickets and prevented early declines in growth in children with X-linked hypophosphataemia [239].
NCT02163577	Completed	XLH	Burosumab	Phase 2	Burosumab improved renal tubular phosphate reabsorption, serum phosphorus levels in children with X-linked hypophosphatemia [243].
NCT02750618	Completed	XLH	Burosumab	Phase 2	Treatment with Burosumab had a favorable safety profile, increased serum phosphorus, and improved rickets and prevented early declines in growth in children aged 1–4 years with X-linked hypophosphataemia [244].
NCT02915705	Completed	XLH	Burosumab, oral phosphate supplement, active vitamin D	Phase 3	Significantly greater clinical improvements in rickets severity, growth among children with X-linked hypophosphataemia treated with Burosumab versus conventional therapy [194].
NCT03920072	Recruiting	XLH	Burosumab	Phase 3	Ongoing
NCT04695860	Recruiting	XLH	Burosumab	Phase 3	Ongoing
NCT04320316	Active, not recruiting	Epidermal nevus syndrome	Crysvita	Phase 4	Ongoing

Note: NAFLD- Nonalcoholic Fatty Liver Disease; NASH- Nonalcoholic Steatohepatitis, T2DM- Type 2 Diabetes Mellitus, XLH- X-Linked Hypophosphatemia.

## 8. Future Perspectives

Endocrine FGFs possess vast potential for developing therapeutics for many different metabolic disorders. Metabolic disease is characterized as a cluster if variable clinical symptoms that occur together, increasing the risk of cardiovascular disease, type 2 diabetes, obesity. Whereas mitochondrial myopathies, are primarily diseases caused by defects in the mitochondria leading to prominent muscular and neurological problems. In most cases, mitochondrial disease is a multi-system disorder affecting more than one type of cell, tissue, or organ. Mitochondrial dysfunction is not only a hallmark of mitochondrial disorders but has also been impli-cated in metabolic disorders in adults. Thus, designing novel small molecules for mitochondrial or metabolic disorders could result in altering disease progression and severity, as well as provide a deeper understanding of how metabolic pathways are rewired in sickness and in health. In recent years, few molecules have emerged attractive therapeutic targets for a range of diseases, spurring active drug discovery efforts in this area. A few examples have been listed below.

MicroRNAs (miRs) are small, noncoding RNAs that function in RNA silencing and post-transcriptional regulation of gene expression [246]. miRs are aberrantly expressed in human diseases with one study providing evidence to demonstrate a link between elevated miR-34a and impaired KLB/FGF19 signaling in obese patients [246,247]. Specifically, mir-34a impaired FGF19/21 signaling in obesity by downregulating the levels of KLB, altering the downstream signalings of ERK and glycogen synthase kinase, and decreasing the expression of FGF19 target genes [247]. Therefore, targeting miR-34a may provide an effective therapeutic option in the treatment of obesity [247].

Mitokines are defined as diffusible molecules like cytokines released by cells or tissues in response to mitochondrial stress, which has beneficial effects on other cells or tissues [248]. FGF21 is considered a mitokine whose expression is modulated in different mitochondrial diseases [126,249]. Thus, FGF21 is considered a biomarker of mitochondrial dysfunction, and alterations of mitokine secretion are reported in many diseases [250]. Interestingly, in a recent study, it was found that high levels of FGF21 mitokine in COVID-19 patients correlates with COVID-19 disease severity and mortality [251]. Therefore, additional studies are required to examine whether modulation of mitochondrial function with mitokine like FGF21 could be a potential therapeutic strategy for COVID-19 patients.

Recent reports on FGF23 have shown that in addition to its role in phosphate reabsorption and vitamin D metabolism, FGF23 plays a role in innate immune and hemodynamic responses [138,252,253,254,255]. Maladaptive excessive FGF23 levels have been observed with inflammation, infection, and cardiovascular diseases, especially in patients suffering from CKD [254,255,256]. Additionally, FGF23 is also induced in macrophages and tissues that do not normally express FGF23 via inflammatory mediators [257,258]. FGF23 is thought to be able to modulate immune functions through indirect and direct pathways [253,258,259]. These new findings open additional possibilities for the development of pharmacological compounds that specifically inhibit the adverse effects of excessive FGF23 levels in patients with CKD [253,260].

In addition, if these compounds could specifically target gut-related FGF19 deficiency or modulate bile acid salts in the digestive system then one could discover new pathways for modulating energy which will provide valuable insight in combatting metabolic disorders. A recent discovery of the key roles played by gut microbiota in an interorgan cross-talk [261] and their development of metabolic diseases [262] opens new and exciting opportunities for understanding the role(s) of the microbiome-to-host signaling pathways, the pathophysiology of diseases, and potential targets for disease intervention. To better understand the link between gut microbiota, bile acid, glucose, lipid or phosphate metabolism, large scale metagenomics, metaproteomics, and metabolomics coupled with deep learning approaches distinguishing healthy and diseased populations could provide precise gut microbial fingerprints to reliably predict the risk of the disease and health of the patient over time. This information could contribute to targeted therapy and novel dietary options such as probiotics could be explored to help restore bile acid homeostasis and aid in the treatment of many metabolic disorders.

In conclusion, the past years have established endocrine FGFs as crucial regulators for many biological processes. Since their discoveries in the early 2000s, there has been a continuous expansion of studies investigating a vast number of topics from biochemistry and structural studies to physiology and pharmaceutical applications of endocrine FGFs. Although many areas of endocrine FGFs have been established, other areas of interest regarding the endocrine subfamily remain unknown and warrant further examinations. Indeed, one can confidently anticipate several new and exciting clinical applications involving endocrine FGFs are in the offing in the near future—The saga of research on these endocrine acting growth factor families continues.

## Abbreviations

1α: 25-dihydroxyvitamin D3, 1,25(OH)2D3; Activating transcription factor 4, ATF4; Adenosine triphosphate, ATP; α-fetoprotein, AFP; AMP-activated protein kinase, AMPK; Angiotensin II, ATII; ASGH, Fetiun A; Autosomal dominant hypophosphatemic rickets, ADHR; cAMP-response element-binding protein-peroxisome proliferator-activated receptor-γ co activator 1α, CREB PGC-1α; Central nervous system, CNS; Cholecystokinin, CCK; Cholesterol-7α-hydroxylase, CYP7A1; Chronic hemodialysis, CD; Chronic kidney disease, CKD; c-Jun N-terminus kinase, JNK; Erythropoietin, EPO; Eukaryotic initiation factor 4B, eIF4B; Extracellular region of human β-klotho, sKLB; Extracellular signal-regulated protein kinase, ERK; Familial tumoral calcinosis, FTC; Farnesiod X receptors, FXR; Farnesoid X receptor ligand obeticholic acid in NASH treatment, FLINT; FGF21 C-terminus, FGF21CT; Fibroblast activation protein, FAP; Fibroblast growth factors, FGFs; Fibroblast growth factors receptors, FGFR; Forkhead box protein O1, FOXO1; Glucose transporter 1, GLUT1; Glycogen synthase, GS; Glycogen synthase kinase 3, GSK3; Glycoside hydrolase 1, GH1; Heparan sulfate proteoglycan, HPSG; Heparin-binding site, HBS; Hepatocellular carcinoma, HCC; High-density lipoprotein, HDL; Human brain vascular smooth muscle cells, hBVSMCs; Insulin-like growth factor, IGF1; Jumonji domain-containing protein D3, JMJD3; Liver X receptor, LXR; Lung squamous cell carcinoma, LSQ; Mechanistic target of rapamycin, mTOR; Mechanistic target of rapamycin complex 1, mTORC1; Mesenchymal stem cells, MSC; Mitochondrial myopathy, MM; Mitogen-activated protein kinase, MAPK; Non-alcoholic fatty liver disease, NAFLD; Non-alcoholic steatohepatitis, NASH; Obeticholic acid, OCA; Parathyroid hormone, PTH; Peroxisome proliferator-activated receptor, PPAR; Phosphoinositide 3-kinase, PI3K; Polyethylene glycol-modified, PEGylated; Protein kinase B, PKB/Akt; Reactive oxygen species, ROS; Receptor-binding arm, RBA; Receptor tyrosine kinase, RTK; Ribosomal protein subunit 6, rpS6; Sodium-dependent phosphate, NaPi; Tissue non-specific alkaline phosphatase, TNAP; Triglyceride, TG; Triosephosphate isomerase, TIM; Tumor-induced osteomalacia, TIO; Type 2 diabetes mellitus, T2DM; Ventromedial hypothalamus, VMH; X-linked hypophosphatemia, XLH; α-klotho, KLA; β-klotho, KLB; 3-hydroxybutyrate dehydrogenase type 1, BDH1; carnitine palmitoyltransferase 1α, CPT1A; glucose-6-phosphatase, G6P; phosphoenolpyruvate carboxykinase, PEPCK.
